# Tumor cell-specific Serpin A1 expression in vulvar squamous cell carcinoma

**DOI:** 10.1007/s00404-018-5015-y

**Published:** 2019-01-04

**Authors:** Maria Lagerstedt, R. Huotari-Orava, R. Nyberg, L. Nissinen, M. Farshchian, S.-L. Laasanen, E. Snellman, J. U. Mäenpää, V.-M. Kähäri

**Affiliations:** 1 Department of Dermatology, Tampere University Hospital, PO Box 2000, 33521 Tampere, Finland; 2 Faculty of Medicine and Life Sciences, University of Tampere, Tampere, Finland; 3 Fimlab Laboratories, Tampere, Finland; 4 Department of Obstetrics and Gynecology, Tampere University Hospital, Tampere, Finland; 5 Department of Dermatology, University of Turku and Turku University Hospital, Turku, Finland; 6 MediCity Research Laboratory, University of Turku, Turku, Finland; 7 FICAN West, University of Turku and Turku University Hospital, Turku, Finland

**Keywords:** Lichen sclerosus, Vulvar squamous cell carcinoma, Serpin A1, p53, p16

## Abstract

**Purpose:**

The two main etiological factors for vulvar squamous cell carcinoma (vSCC) are the vulvar dermatosis lichen sclerosus (LS) and high-risk human papillomavirus (hrHPV). Serpin A1 (α1-antitrypsin) is a serine protease inhibitor, which plays a role in the tumorigenesis of various cancer types. The aim of the study was to evaluate the expressions of Serpin A1 in LS, premalignant vulvar lesions, and vSCC using immunohistochemistry (IHC) and serum analysis, and to compare Serpin A1 stainings to the tumor markers p53 and p16.

**Methods:**

In total, 120 samples from 74 patients were studied with IHC for Serpin A1, p53 and p16: 18 normal vulvar skin, 53 LS, 9 premalignant vulvar lesions (dVIN/HSIL) and 40 vSCC samples. Serum concentrations of Serpin A1 were analyzed from 30 LS, 44 vSCC and 10 control patients. Expressions were compared to clinical data.

**Results:**

Tumor cell-specific Serpin A1 overexpression was detected in 88% of vSCC samples, independent of the etiology. The intensity of Serpin A1 expression was significantly higher in vSCC than in healthy vulvar skin, LS, or premalignant vulvar lesions. Serpin A1 showed an association with p53 positivity. No difference in overall survival was found between Serpin A1-, p53-, or p16-positive vSCC patients. Serum concentrations of Serpin A1 were equal in the LS, vSCC, and control groups.

**Conclusion:**

Tumor cell-specific Serpin A1 overexpression is a potential biomarker in vSCC.

## Introduction

Vulvar squamous cell carcinoma (vSCC) accounts for approximately 5% of the tumors of the female genital tract and has two main etiologies: high-risk human papillomavirus (hrHPV) and the chronic vulvar dermatosis lichen sclerosus (LS) [1]. LS-dependent vSCC typically affects elderly women (on average, 60–80 years of age), while HPV-dependent vSCC occurs at a younger age and is associated with the number of sexual partners, smoking, and immunological deficiencies [2]. These two pathways differ from each other by their precursor lesions, which are differentiated vulvar intraepithelial neoplasia (dVIN) for LS-dependent and high-grade squamous intraepithelial lesions (HSIL, previous WHO nomenclature usual VIN2-3) for HPV-dependent vSCC, but also by their genetic alterations and protein markers [3]. *TP53* mutations, which are detectable by means of immunohistochemical (IHC) methods, showing either a p53-positive or p53-null staining pattern, are considered as a hallmark of the carcinoma process related to LS [2, 3]. On the other hand, intensive p16 staining is a surrogate marker of hrHPV infection [4]. The difference in survival between these two groups has remained controversial [1, 2]. However, the pathways overlap with respect to their clinical and histological features, as well as their IHC findings. Overall, the understanding of the pathogenetic mechanisms of malignant progression especially in LS-dependent SCC is limited, and biomarkers are warranted for clinical use [5].

Serpin peptidase inhibitor clade A member 1 (Serpin A1), also called α1-antitrypsin, is a serine protease inhibitor [6]. Serpin A1 is an acute-phase protein that effectively inhibits the activity of neutrophil elastase, and also that of, for example, trypsin, chymotrypsin, plasmin, and thrombin [7]. Serpin A1 plays a role in blood coagulation, angiogenesis, and the remodeling of the extracellular matrix [8]. Various tumor cells also produce Serpin A1 [6]. Serpin A1 has been shown to exert an anti-apoptotic and tumor-promoting effect and is considered a marker for poor prognosis in gastric, lung, colorectal, ovarian, and cervical cancer [6, 9–12]. Overexpression of the Serpin A1 gene is associated with cutaneous SCC and the progression of esophageal squamous dysplasia, and plasma levels of Serpin A1 are elevated in esophageal SCC as well as gastric, prostate, lung, and colorectal cancer [13–19].

The expression of Serpin A1 is associated with various cancer types, including SCC, but has not previously been studied in vSCC. The aim of our study was to evaluate the role of Serpin A1 in the malignant progression of vSCC using IHC and serum analysis, as well as to compare the Serpin A1 expressions to tumor markers p53 and p16 in vSCC.

## Materials and methods

The Ethics Committee of Tampere University Hospital approved the study protocol (R11026) and the Finnish National Supervisory Authority for Welfare and Health gave its official permission for the use of diagnostic histological LS and vulvar SCC samples (D9050/06.01.03/2013). Serum samples and healthy vulvar skin biopsies were obtained from volunteer patients being treated at the Department of Gynecology and Obstetrics of Tampere University Hospital. All volunteers gave their informed consent for the study. The patient records were accessed with the permission of the medical director of Tampere University Hospital to verify the course and staging of the disease.

### Patients and samples

The study included a total of 120 biopsy and vulvectomy samples from LS and vSCC patients treated at the Department of Gynecology or the Department of Dermatology at Tampere University Hospital in 2006–2008 and 2010–2013.

Healthy vulvar skin tissue samples were obtained from 18 volunteer vSCC patients and the biopsies were taken from healthy-looking skin in the vulvar area. The study included 30 patients with vulvar LS without malignant progression (aged 42–86 years, median 71 years) and 40 patients with vSCC (aged 39–90 years, median 72 years). LS was histologically verified by dermatopathologist R.H-O in 24 (60%) of vSCC patients. No other vulvar dermatoses were detected in the vSCC patients. The majority of the LS samples without malignant progression were diagnostic (26/30) and were taken before potent corticosteroid treatment. Only three LS patients with malignant progression were on potent corticosteroid treatment prior to the biopsies. Six HSIL and three dVIN samples surrounding the carcinoma area were also available. The clinical data, including FIGO staging and lymph node status, the histological grade of the tumor, and recurrence time, were available for all vSCC patients, and data on the tumor size and invasion depth were available for 34 of the vSCC patients.

Serum samples were collected from 84 volunteers: 29 patients with vSCC before surgical treatment, 15 surgically treated vSCC patients during follow-up (on average 2.6 years after surgery), 30 LS patients and 10 age-matched uterine prolapse patients serving as controls. Patients with a history of any other tumors and those with an acute infection or other inflammatory diseases were excluded.

### Immunohistochemical studies

All tissue samples were formalin-fixed paraffin-embedded. The Serpin A1 stainings were performed with the polyclonal rabbit anti-human Serpin A1 antibody (ATT, code no A0012, DakoCytomation) using an automated immunostaining device (Ventana Medical Systems SA, Illkirch, France) and the Ventana UltraView Universal DAB detection kit and the Ventana amplification kit (Ventana Medical Systems SA). The dilution used was 1:7000. Liver tissue was used as a positive control.

p53 and p16 stainings were performed with prediluted mouse monoclonal antibodies anti-p53 (Bp53-11, cat.nro. 760-2542, Ventana Medical Systems, Inc.) and CINtec p16 histology (E6H4, cat.nro. 725-4713, Ventana Medical Systems, Inc.) using a Ventana BenchMark immunostainer and Ventanan Ultraview DAB Detection Kit (Ventana, Tucson, Arizona) according to the manufacturer’s instructions. Ultrablock antibody diluent (cat.nro. 251-018, Ventana Medical Systems, Inc.) was used for p16 staining. High-grade serose adenocarcinoma and cervical carcinoma in situ were used as positive controls for p53 and p16, respectively.

All samples were analyzed independently by a dermatopathologist (R.H-O) and author 1. Cytoplasmic staining of Serpin A1 was considered specific and the staining intensity was scored as follows: negative (0), weak (+), moderate (++), and strong/positive control (+++). The staining intensity was evaluated from the most invasive area of the tumor.

In our study p53 staining was scored < 1% or null, 1–10% or normal, 10–50% and > 50% of the tumor cell nuclei positive, but only more than 50% of the tumor nuclei stained was considered positive for statistical analysis, in accordance with clinical practice. In LS, both continuous and discontinuous basal epidermal keratinocyte staining was considered positive. Less than 10% nuclei positivity in LS was considered negative in accordance with normal vulvar skin where single epidermal keratinocytes and melanocytes stain.

Staining for p16 was interpreted as positive when over 75% of the cells showed intensive cytoplasmic and nuclear staining, i.e., so-called “block positivity.”

### Serum sample preparation and analysis of Serpin A1 serum levels

The serum samples were collected into Venosafe serum tubes, centrifuged 2000*g* for 10 min and stored in cryotubes at − 70 °C until use. An immunonephelometric assay (BM ProSpec automatic analyzer, Siemens Healthcare Diagnostics Inc., Siemens Aktiengesellschaft, Munich, Germany) was used for the analysis of Serpin A1 serum concentrations.

### Statistics

Pearson’s Chi square test was used to compare the Serpin A1 staining intensity between different patient groups. Fisher’s exact test was used to determine the association between Serpin A1 staining and categorical variables (histological grade, FIGO staging, and immunopositivity of p53 and p16). Kaplan–Meier analysis was used to compare the overall survival in LS-, p53- and p16-positive and negative vSCC patients, and in the comparison of Serpin A1 intensity and survival.

## Results

**Fig. 1:**
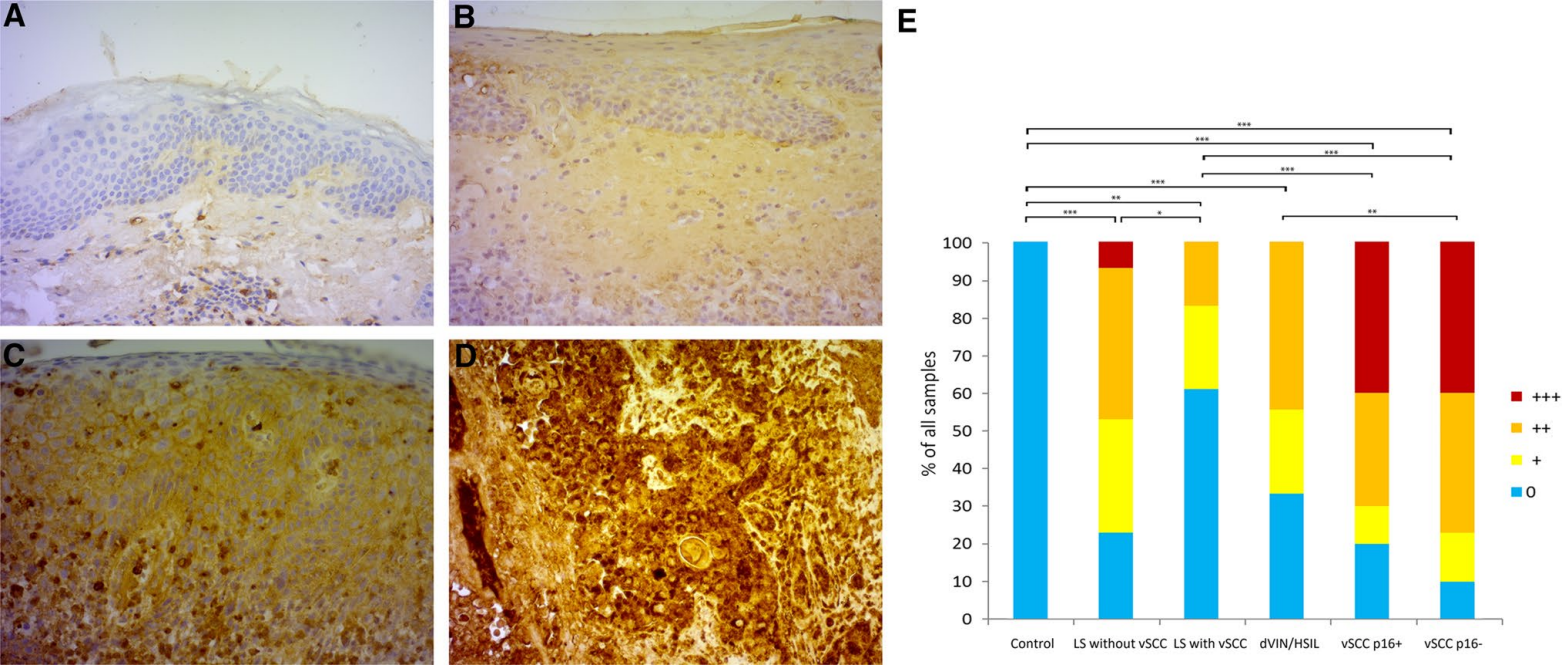
Expressions of Serpin A1 in vulvar skin, lichen sclerosus (LS), differentiated vulvar intraepithelial lesions/high-grade squamous intraepithelial lesions (dVIN/HSIL), and vulvar squamous cell carcinoma (vSCC). **a** The epidermal layer of healthy vulvar skin is negative for Serpin A1. **b** Weak (+) cytoplasmic staining of the epidermis in LS, **c** moderate-intensity (++) staining of dVIN, and **d** strong (+++) tumor cell-specific staining in vSCC. **e** Semiquantitative analysis of Serpin A1 staining in healthy vulvar skin (*n* = 18), LS without vSCC (*n* = 30), LS with vSCC (*n* = 23), dVIN/HSIL (*n* = 9), p16-negative vSCC (*n* = 30) and p16-positive vSCC (*n* = 10). ***p < 0.001, **p < 0.01, *p < 0.05 (*χ*^2^ test). Original magnifications × 200

The epidermal layer of healthy vulvar skin was negative for Serpin A1 staining (Fig. 1a). Of all the 53 LS samples 40% were negative, whereas 26% showed weak (+) (Fig. 1b) and 30% moderate (++) cytoplasmic Serpin A1 staining. Only 4% of the LS samples showed strong (+++) staining. LS without malignant progression showed more intensive Serpin A1 staining than LS samples from vSCC patients (*p* = 0.024*). Four LS patients without malignant progression had used potent corticosteroid treatment prior the biopsy, and the biopsy samples of three of them showed negative staining for Serpin A1. There was a statistically significant correlation between negative staining for Serpin A1 and treatment with potent corticosteroids in this patient group (*p* = 0.01**). In LS samples from vSCC patients the correlation between Serpin A1 staining and corticosteroid treatment was not detected. The staining intensity in premalignant vulvar lesions was equal in HSIL and dVIN samples and showed weak or moderate staining (Fig. 1c) in 67% of the samples, being negative for Serpin A1 in 33% of the samples. The majority (75%) of the 40 vSCC samples showed tumor cell-specific strong (Fig. 1d) or moderate cytoplasmic staining for Serpin A1, and weak tumor cell-specific staining was detected in 13% of the vSCC samples. The expression of Serpin A1 was significantly higher in vSCC than in healthy vulvar skin, LS, or HSIL/dVIN samples (Fig. 1a–e). Serpin A1 staining intensity did not correlate with the histological grade of the tumor, the FIGO staging, the recurrence, the lymph node status, the depth of tumor invasion (< 4 mm vs. > 4 mm), or the tumor size (< 40 mm vs. > 40 mm) of the vSCC patients.

**Fig. 2:**
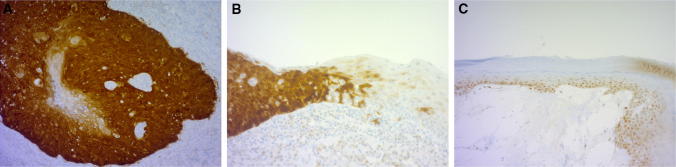
Expression of tumor marker p16 in vSCC and LS. **a** Intensive nuclear and cytoplasmic p16 positivity in vSCC; **b** p16 positivity in transition to HSIL; and **c** mosaic/mild epidermal p16 staining in LS adjacent to p16 positive vSCC, interpreted as p16 negative. Original magnifications × 200

Positive p16 staining, indicating hrHPV positivity, was detected in 10 (25%) of the vSCC samples and 50% of HSIL samples (Table 1, Fig. 2a, b). LS and p16 positivity coexisted in three out of all 40 vSCC cases (8%). All LS samples were interpreted as negative for p16, but mild/mosaic basal epidermal p16 staining was already apparent in LS samples from next to a p16-positive tumor (Fig. 2c).

**Table 1:** p53 and LS status in both p16-positive and p16-negative vulvar squamous cell carcinoma (vSCC), high-grade intraepithelial lesion (HSIL) and differentiated vulvar intraepithelial neoplasia (dVIN) tissue samples

Samples	vSCC, p16+ *n* = 10	vSCC, p16− *n* = 30	HSIL *n* = 6	dVIN *n* = 3
p53-positive (%)	3 (30%)	17 (57%)	3 (50%)	1 (33%)
p53-null (%)	4 (40%)	5 (17%)	2 (33%)	1 (33%)
LS-positive (%)	3 (30%)	27 (90%)	2 (33%)	3 (100%)

p53 data not available from two p16-negative vSCC patients

*LS* lichen sclerosus

**Fig. 3:**
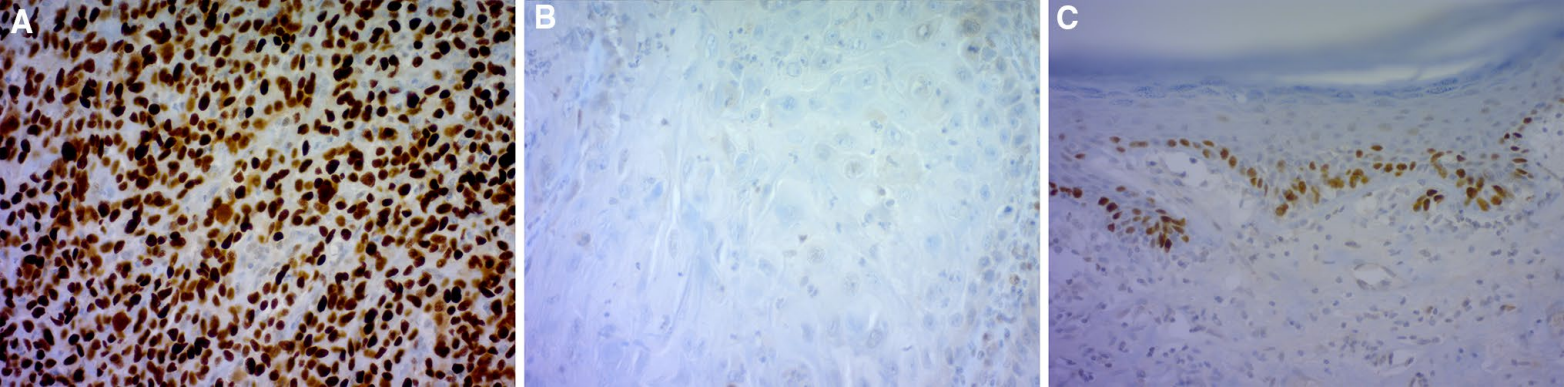
Expression of tumor marker p53 in vSCC and LS. **a** p53-positive staining (over 50% of tumor nuclei stained) in vSCC; **b** p53-null staining (< 1% of tumor nuclei stained) in vSCC; and **c** p53-positive “band-like” staining in the nuclei of basal epidermal keratinocytes in LS. Original magnifications × 200

Positive p53 staining was detected more frequently in p16-negative (57%) than p16-positive vSCC (30%) samples, and p53-null staining was detected in 23% of the vSCC samples (Table 1, Fig. 3a, b, respectively). For tissue samples from two vSCC patients p53 data were not available. The LS samples from vSCC patients showed continuous or discontinuous “band-like” p53 positivity in basal epidermal cells in 35% of the samples, while LS without malignant progression was p53-positive in 93% of the cases, and the difference was statistically significant (*p* < 0.001***) (Fig. 3c). Six (15%) of the 40 vSCC samples were negative for both p53 and p16, and of four (67%) out of the six samples showed strong or moderate Serpin A1 staining. There was a statistically significant correlation between p53 positivity and Serpin A1 positivity (*p* = 0.017*) in vSCC, but Serpin A1 and p16 positivity showed no association. No statistically significant difference in overall survival was observed in vSCC patients with and without LS, but the mean survival was better in the LS-negative vSCC patient group (6.3 years vs. 5.6 years, respectively). The overall survival of our vSCC patients showed no correlation with Serpin A1 intensity, nor with p53 or p16 positivity. The average follow-up time was 8.1 years (range 3.2–11.0 years) from diagnosis to the end of the study period with exitus in 25/40 (63%) of the patients.

No statistically significant differences were detected in Serpin A1 serum concentrations between the active vSCC, treated vSCC, LS or control groups. The mean respective values and ranges were 1.483 g/l (0.893–2.05 g/l), 1.463 g/l (1.17–1.77 g/l), 1.438 g/l (0.95–1.78 g/l) and 1.472 g/l (1.15–2.01 g/l). The normal range for Serpin A1 serum concentrations is 0.96–1.78 g/l.

## Discussion

To our knowledge, this was the first study showing tumor cell-specific Serpin A1 overexpression in vSCC. The expression of Serpin A1 was significantly increased in vSCC compared to healthy vulvar skin, LS, and premalignant vulvar lesions. The overexpression of Serpin A1 was independent of the etiology of vSCC and, therefore, connects the two inflammatory etiologies of vSCC. Positivity of p53 was associated with Serpin A1 overexpression in vSCC, but no correlation was detected between Serpin A1 and the histological grade of the tumor, the FIGO staging or the survival of our patients.

Serpin A1 overexpression is a marker of poor prognosis in various cancer types [6, 9–13, 15, 20]. Primary tumor cells and metastases produce Serpin A1, and elevated plasma concentrations have been detected in SCC of the esophagus and oral cavity, among others [15–19, 21]. Our study revealed no difference in Serpin A1 serum concentrations between controls, LS, and vSCC patients. Due to the physiological function of Serpin A1 as an acute-phase protein, its serum concentration may thus not be sensitive enough for the evaluation of malignant progression in vSCC.

Several tumorigenic mechanisms of Serpin A1 have been identified. Serpin A1 mediates anti-apoptotic effects through the TNFα-signaling pathway, but also directly via blocking caspase-3 activity [22]. In addition, in gastric, ovarian, breast, colorectal, and lung cancer cells, Serpin A1 promotes tumor cell migration and invasion capacity [6, 10, 11, 23, 24]. Serpin A1 has a role in angiogenesis, complement activation and the remodeling of the extracellular matrix, thus making it a factor of the tumor microenvironment [8]. Even though there is an increasing understanding of the role of chronic inflammation and the tumor microenvironment in cancer progression, previous studies on the vSCC tumor microenvironment are limited [25, 26]. The present study found a novel agent, Serpin A1, in the microenvironment of vSCC.

Serpin A1 plays a role in the regulation of inflammation and autoimmunity [7]. Serpin A1 expression is enhanced by the cytokines EGF, TNF-α, INF-γ, and IL-1β in cutaneous SCC cells and the latter three are also upregulated in lichen sclerosus [13, 26]. In other autoimmune diseases, such as SLE, rheumatoid arthritis, and diabetes mellitus type 1, the role of Serpin A1 is anti-inflammatory and cytoprotective, which may explain the more abundant Serpin A1 expression in LS without malignant progression [7]. The treatment with potent corticosteroid ointments also seemed to correlate with negative Serpin A1 staining in LS without malignant progression. This may suggest, as noted by Lee et al. [27], that effective treatment of LS with potent corticosteroid ointments can minimize the risk of malignant progression.

In our study, the expression of Serpin A1 increased gradually from LS to premalignant vulvar lesions to vSCC. Serpin A1 has been shown to interact with NF-κB–TNFα-axis, an important pathway in immunology and inflammatory cancers which also associates with both HPV-infection and lichen sclerosus [7, 25, 28]. Therefore, Serpin A1 may promote tumorigenesis in both pathways to vSCC.

We found a correlation between Serpin A1 overexpression and p53 positivity in our vSCC samples. A recent study on lung cancer shows that mutant p53 upregulates Serpin A1 expression and promotes tumor invasion [29]. A strong association between LS and the LS-dependent vSCC process and *TP53* mutations has been reported in previous studies [1–3]. In line with this, we observed basal epidermal p53-positivity in 67% of all LS samples and as frequently as in 93% of LS samples without malignant progression [30, 31]. This supports the notion that p53 positivity in LS is linked with ischemic stress and repairing mechanisms against stress rather than with the malignant potential of LS [31]. In our study, 50% vSCC samples showed positive staining for p53, which is in line with earlier studies [1, 5]. Nonsense mutations of *TP53* can lead to the absence of the p53 protein (so-called p53 null), or the p53 protein can be degraded by HPV oncoprotein E6 [1, 3]. As many as 9/40 (23%) of our vSCC samples represented this p53-null type. Our results highlight the essential role of p53 in the malignant process of vSCC.

Interestingly, as many as 6/40 (15%) of the vSCC samples were both p53- and p16-negative. The majority of these samples (67%) showed strong or moderate Serpin A1 staining. A previous study by Nooij et al. has found this third subgroup of vSCC to harbor mutations in the NOTCH1 and HRAS genes [32]. Therefore, the etiopathology of these p53- and p16-negative tumor types may be variable and constitutes an interesting field for future studies.

The overall survival of our vSCC patients did not correlate with Serpin A1, p16 or p53 positivity, but a worse prognosis seemed to associate with the presence of LS. However, the number of patients in the study and also follow-up time were limited for survival analysis. As an interesting notion of study population, there were three vSCC patients with coexisting LS and p16 positivity. Testing for hrHPV could therefore be beneficial to LS patients to keep those patients with two risk factors for vSCC under a more intensive surveillance.

## Conclusion

This study found a novel biomarker, Serpin A1, to be associated with vSCC independent of the etiology. Serpin A1 serum concentrations or IHC showed no prognostic value in LS or vSCC patients. However, better understanding of the vSCC microenvironment may provide new avenues for cancer therapy studies in the future.
